# Variation in the cortical area map of C57BL/6J and DBA/2J inbred mice predicts strain identity

**DOI:** 10.1186/1471-2202-6-18

**Published:** 2005-03-17

**Authors:** David C Airey, Alicia I Robbins, Katherine M Enzinger, Fangbai Wu, Christine E Collins

**Affiliations:** 1 Department of Pharmacology, Vanderbilt University Medical Center, Nashville, TN, USA; 2 Department of Psychology, Vanderbilt University, Nashville, TN, USA

## Abstract

**Background:**

Recent discoveries suggest that arealization of the mammalian cortical sheet develops in a manner consonant with principles established for embryonic patterning of the body. Signaling centers release morphogens that determine regional growth and tissue identity by regulating regional expression of transcription factors. Research on mouse cortex has identified several candidate morphogens that affect anteroposterior or mediolateral cortical regionalization as well as mitogenesis. Inbred strains of laboratory mice can be exploited to study cortical area map formation if there are significant phenotypic differences with which to correlate gene polymorphism or expression data. Here we describe differences in the cortical area map of two commonly used inbred strains of laboratory mice, C57BL/6J and DBA/2J. Complete cortical hemispheres from adult mice were dissected and stained for the cytochrome oxidase enzyme in order to measure histochemically defined cortical areas.

**Results:**

C57BL/6J has the larger neocortex, relatively larger primary visual cortex (V1), but relatively smaller posterior medial barrel subfield of the primary somatosensory cortex (PMBSF). The sample of C57BL/6J and DBA/2J mice can be discriminated with 90% accuracy on the basis of these three size dimensions.

**Conclusion:**

C57BL/6J and DBA/2J have markedly different cortical area maps, suggesting that inbred strains harbor enough phenotypic variation to encourage a forward genetic approach to understanding cortical development, complementing other approaches.

## Background

Species differences in cortical regionalization reflect genetic and epigenetic developmental programs that are presumed adaptations to different ecological niches. For example, Krubitzer and Kahn [1] review that the mouse, ghost bat, and short-tailed opossum have approximately the same size cortical sheet, but differ substantially in the size of one of three primary sensory cortical areas. The enlarged cortical area in each species reflects a greater behavioral reliance on the represented sensory modality. In the same sensory modality, functional specialization of the sensory periphery is also reflected in the primary cortical area size. Catania and Remple [2] show that the naked mole-rat, a fossorial species dependent on somatosensation, has a much greater cortical surface area devoted to somatosensation compared to the laboratory rat. As discussed by Krubitzer and Kahn [1], although differences in genetic determination are well implicated by these species differences, identity of the genes and their developmental actions are not well understood.

Recent experimental manipulations in mice have caused striking qualitative and quantitative changes in the cortical area map, identifying several candidate morphogens that affect anteroposterior or mediolateral cortical regionalization as well as mitogenesis [3]. For example, Fukuchi-Shimogori and Grove [4] placed an ectopic caudal source of fibroblast growth factor 8 into the developing mouse cortex and caused a caudal duplication of part of the primary somatosensory area. Hamasaki et al. [5] demonstrated changes in the position and or size of primary sensory visual, somatosensory, and auditory cortical regions in transgenic mice over expressing the transcription factor *Emx2*. These and other discoveries (reviewed by [1,3]) suggest that arealization of the mammalian cortical sheet develops in a manner consonant with principles established for embryonic patterning of the body. Signaling centers release morphogens that determine regional growth and tissue identity by regulating regional expression of transcription factors. Grove and Fukuchi-Shimogori [3] note that such research has provided a starting point for investigating how the cortical area map is generated and modified in a single individual and how maps change in the course of evolution, but that a major step forward would be to identify novel transcription factors involved in cortical area patterning. Along these lines, Funatsu et al. [6] have employed gene expression array analysis of the dissected embryonic (16.5d) mouse cerebral cortex to expand the list of genes regionally expressed, and noted that regional differences in expression of genes in the cortical plate should eventually convert into functionally distinct cortical areas with anatomically distinguishable borders after birth.

Inbred strains of laboratory mice can be exploited to study and understand complex traits of the nervous system if there is significant phenotypic variation with which to correlate gene polymorphism [7-9] or expression data [10]. As the first step to an integrative and relational discovery program [11,12] in a model system for mammalian cortical area map formation, here we describe significant differences in the cortex of two common inbred strains of laboratory mice, C57BL/6J and DBA/2J.

## Results

### Neocortex, visual cortex, and barrel cortex differ between C57BL/6J and DBA/2J

**Figure 1:**
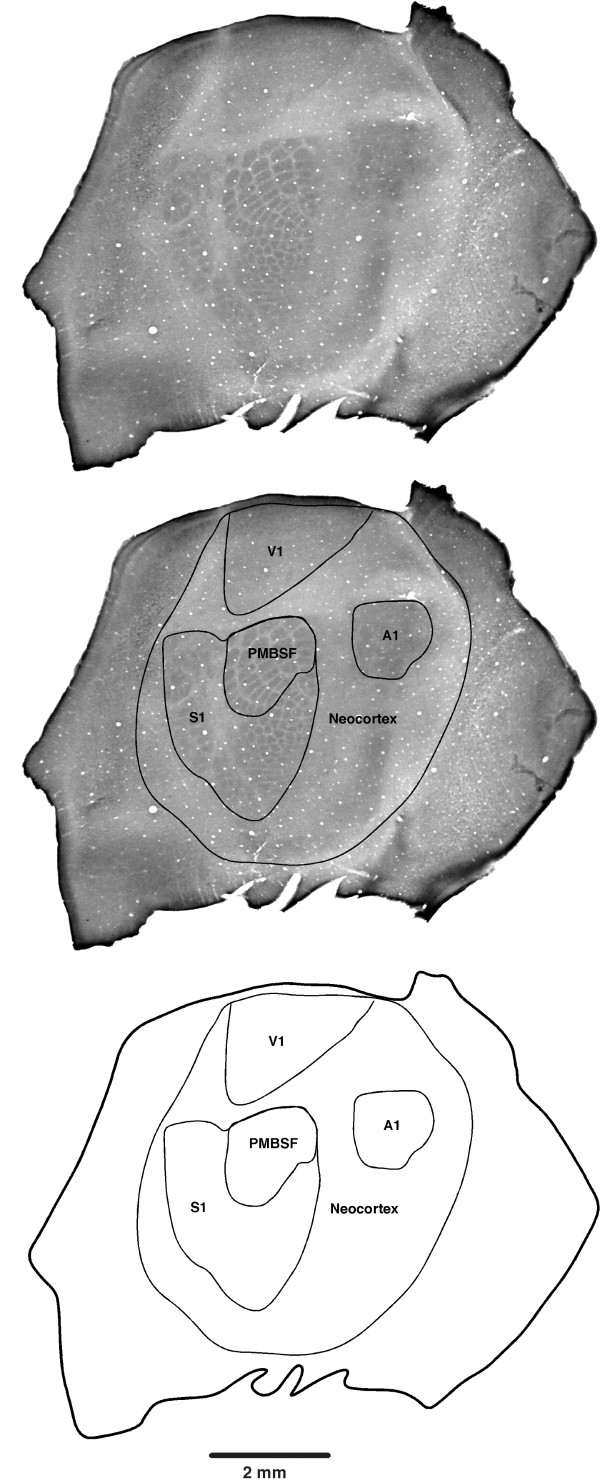
**Flattened mouse cortex stained for cytochrome oxidase**. Figure 1 top to bottom shows a typical flattened section of a mouse cortex stained for cytochrome oxidase and the boundaries of neocortex, visual cortex, barrel cortex (PMBSF), somatosensory cortex, and auditory cortex outlined in black.

**Figure 2:**
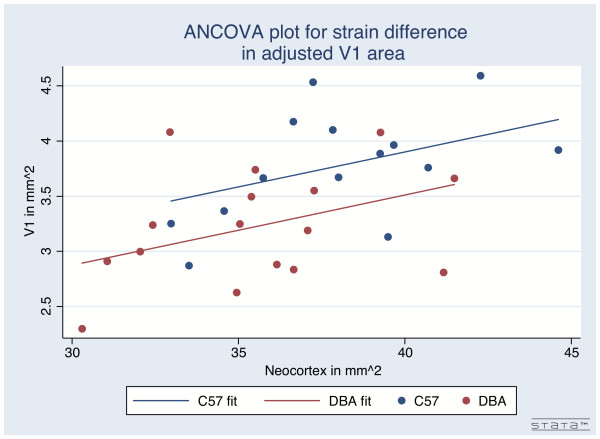
**ANCOVA plot for visual cortex**. Figure 2 shows a significant over representation (distance between parallel lines) of visual cortex in C57BL/6J mice.

**Figure 3:**
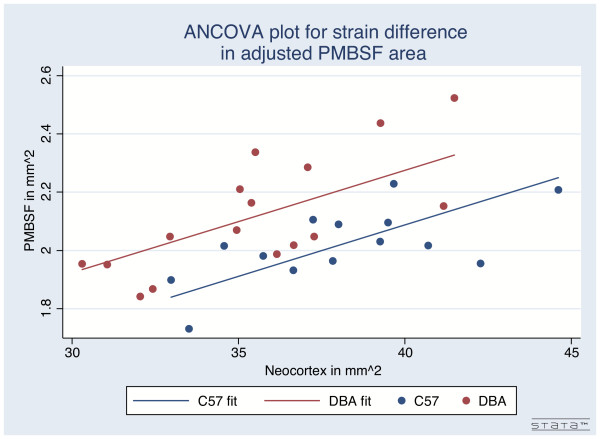
**ANCOVA plot for barrel cortex**. Figure 3 shows a significant over representation (distance between parallel lines) of barrel cortex in DBA/2J mice.

Using established histochemical methods to visualize neocortical and primary cortical areas (see Methods and Figure 1), we estimate that neocortex (C) is on average 7% larger in C57BL/6J (38.0 mm^2^) compared to DBA/2J (35.5 mm^2^) (F_1,28 _= 4.25, P = 0.049). While both visual cortex and barrel (PMBSF) cortex areas significantly correlate with total neocortex area (r_*V*1,*C *_= 0.38, P = 0.039; r_*PMBSF*,*C *_= 0.47, P = 0.009), each also uniquely differs between strains. Using ANCOVA to control for variation in neocortex size, the adjusted mean visual cortex areas for C57BL/6J and DBA/2J are 3.69 and 3.30 mm^2^, respectively, a 12% difference (F_1,27 _= 4.51, P = 0.043). Using ANCOVA, the neocortex adjusted mean barrel cortex areas for DBA/2J and C57BL/6J are 2.16 and 1.97 mm^2^, respectively, a 10% difference (F_1,27 _= 14.72, P < 0.001). These results are shown graphically in Figures 2 and 3. In Figure 2, the ANCOVA plot shows that C57BL/6J mice have more visual cortex; the C57BL/6J linear fit is above the DBA/2J linear fit. In Figure 3, the ANCOVA plot shows that DBA/2J mice have more barrel cortex; the DBA/2J linear fit is now above the C57BL/6J linear fit, reversed from Figure 2. There is no significant evidence for heterogeneity of slopes in the fitted lines from Figure 2 or Figure 3 when interaction terms are added to the ANCOVA models.

### Cortical field size configuration predicts C57BL/6J and DBA/2J strains

**Figure 4:**
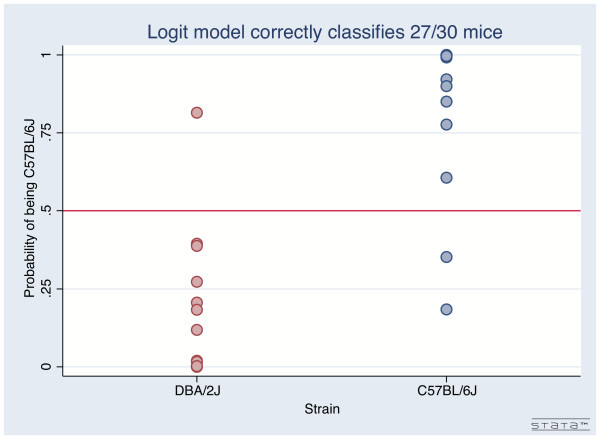
**Prediction plot for strain identity**. Figure 4 plots the predicted probability of each mouse being C57BL/6J by strain from a logistic regression model including neocortex, visual cortex, and barrel cortex areas.

**Figure 5:**
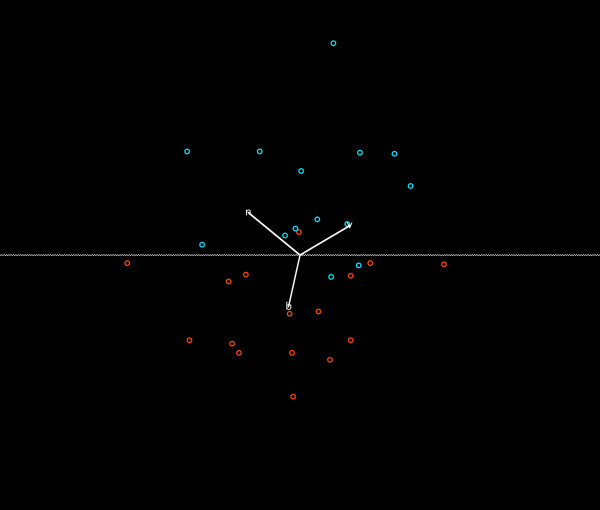
**Projection of a rotating plot**. Figure 5 is a 2-dimensional projection of a 3-dimensional rotating plot of neocortex, visual cortex, and barrel cortex areas (centered by mean and scaled by standard deviation). The projection shows the separation of strains predicted by the logistic regression model (or equivalent discriminant analysis).

Given the significant differences in neocortex, visual cortex, and barrel cortex areas between C57BL/6J and DBA/2J, we can ask how well these measures collectively predict or discriminate strain identity. In a logistic regression model predicting strain identity from neocortex, visual cortex, and barrel cortex areas, the overall model is significant (likelihood ratio *χ*^2 ^(3 df) = 25.45, P < 0.001; Hosmer-Lemeshow goodness-of-fit *χ*^2 ^(8 df) = 1.58, P = 0.99), as is neocortex area (likelihood ratio *χ*^2 ^(1 df) = 10.32, P = 0.001), visual cortex area (likelihood ratio *χ*^2 ^(1 df) = 9.86, P = 0.002), and barrel cortex area (likelihood ratio *χ*^2 ^(1 df) = 17.89, P < 0.001). Table 1 shows the prediction table for this model, revealing 27 out of 30 mice (90%) were correctly classified by strain. Figure 4 and Figure 5 graphically portray these results. Figure 4 plots the predicted probabilities from the logistic regression model by strain. Figure 5 plots a projection from a three dimensional rotating plot for neocortex, visual cortex, and barrel cortex. When rotated to the projection shown, a plane (or line) separates the strains with 90% accuracy.

### C57BL/6J and DBA/2J strains are not differentiated on other dimensions

Neither the area of somatosensory cortex (S1) taken as a whole, nor auditory cortex (A1) area was found to be significantly different between strains. Subjectively, we found the dorsal border of S1 (upper lip, forepaw, hindpaw, and trunk representations) and the borders of A1 to be more difficult to distinguish in our tissue than either V1 or PMBSF. Neither S1 nor A1 area measures added significantly to the ability to predict strain identity.

## Discussion

Grove and Fukuchi-Shimogori [3] and Krubitzer and Kahn [1] suggest that the cortical map, at least for primary areas, develops independently of thalamic input, by way of signaling centers releasing morphogens that determine areal identity. This is not a claim that thalamic projections to the cortex do not play a role in cortical area map formation [1,13], but rather that principles of development observed in body embryogenesis are also active in early cortical area map formation. Developmental and genetic manipulations have produced striking evidence for a handful of candidate morphogens in cortical area map formation [4,5,14]. Recent gene array expression studies are expanding the list of genes that may act to pattern the mammalian cortex [6]. To date, there is little evidence these candidate morphogens cause individual or species differences. An approach that ties candidate or novel morphogens to cortical area map development and anatomy within the range of normal individual differences would prove complementary. Complex trait analysis of the mouse central nervous system [11], allied with gene expression approaches [10], can be used with recombinant inbred strains of mice [15] to provide a cumulative, integrative discovery program [12] that has the potential to tie genomic and transcriptomic variation to variation of the central nervous system and the behaving organism. Here we provide evidence that the cortical area map differs significantly even in two inbred strains of laboratory mice, C57BL/6J and DBA/2J. Importantly, these are the parental strains of the BXD recombinant inbred strains [15], and this study thus provides an empirical basis for using this mammalian neurogenetic resource to study cortical development.

In this paper we have shown that neocortex, visual cortex, and barrel cortex differ in area between C57BL/6J and DBA/2J inbred strains of mice, and that collectively, these measures accurately discriminate these strains. The relatively greater barrel cortex representation in DBA/2J confirms an earlier abstract reporting greater representation of the barrel field in DBA/2J compared to C57BL/6J mice [16]. The larger neocortex in C57BL/6J is consistent with the larger brain size in this strain [8]. The dimension of area is one way to measure the cortical map. Other experimental studies have shown changes in field duplication [4], number [17], or position [5]. In future studies of either BXD recombinant inbred lines, or of other commercial inbred strains [18,19] or their derivatives, we suggest that more powerful statistical descriptions of the size and shape of the cortical area map could be used. Borrowing from advances in the field of geometric morphometrics [20], methods that have been applied to the genetic architecture of the Drosophila wing shape [21], or mouse mandible shape [22], could be applied to the mouse cortical area map, with landmarks defined by classical histochemical or immunohistological stains or by other molecular markers. Landmark-based shape statistics are not a panacea for the measurement of biological form [23], but the point with regard to using isogenic strains is that landmarks can be investigated by replicate measures for reliability and can be correlated with genomic data, or transcriptomic data, at a particular developmental milestone, or across milestones. This is a promising research direction that would complement efforts to answer how the cortex develops, what are the functional or dysfunctional consequences for a particular cortical configuration, and even how the cortex has evolved or can evolve [24,25].

## Conclusion

Inbred strains of laboratory mice can be used to investigate mammalian cortical area map formation if there is significant phenotypic variation with which to correlate gene polymorphism or expression data. Adult C57BL/6J and DBA/2J mice are markedly different in cortical area maps, suggesting that inbred strains harbor enough phenotypic variation to encourage a forward genetic approach to understanding cortical development, complementing other approaches.

## Methods

### Animals

Mice were purchased from Jackson Laboratories at 4–6 weeks of age, housed on a 12:12 light:dark cycle in same sex groups in standard laboratory animal cages (5 animals per cage). All experimental procedures were performed in accordance with the *Guidelines for the Care and Use of Laboratory Animals *published by the National Institutes of Health (publication 86-23) and the Vanderbilt University Animal Care and Use Committee. Mice were provided a standardized diet and clean water ad libitum until they were killed. The cortices of thirty young adult (6–8 weeks of age) mice were measured in this study (14 C57BL/6J, 16 DBA/2J). Mice at this age are not compromised by known visual and or auditory sensorineural deficits common to older animals of these strains. None of the mice used had visible body or facial wounds. A pilot study of 10 older aged C57BL/6J mice indicated drawings of cytochrome oxidase material were reliable for the cortical measures reported here, in that significant animal differences could be detected within strain, and that neither sex nor hemisphere main effects were significant. In the sample of 30 young adult mice reported here, both sexes were sampled, but sex effects were not detected. Hemisphere measures were averaged by animal when two hemispheres were measured. No age effects were associated within the narrow age span sampled. All measurements were made while blind to strain and animal identity.

### Cortex

Mice were brought to complete anesthesia with a sodium pentobarbital overdose (100 mg/kg) injected intraperitoneally (IP), and then transcardially perfused with 0.1 M phosphate buffered 0.9% saline wash followed by 3% buffered paraformaldehyde fixative. Intact brains were removed from the skull, and the cortex was dissected free of the underlying white matter. Dissected cortices were transferred to 30% sucrose, and flattened between glass slides for 12 hours. Cortices were sectioned parallel to the cortical surface at a thickness of 80 *μ*m on a freezing, sliding microtome, stained for cytochrome oxidase according to the method of Wong-Riley [26], mounted on glass slides, air dried, and coverslipped.

### Measurements

The outlines of five regions of interest were drawn under a light microscope with a camera lucida attachment. Regions of interest included neocortex (C), visual cortex (V1), auditory cortex (A1), somatosensory cortex (S1), and the posterior medial barrel subfield (PMBSF) (see Figure 1). Barrel rows A, B, C, D, and E were collectively bounded for the PMBSF, and standardized to 5, 4, 6, 7, and 8 barrels, respectively (alpha, beta, gamma, and delta barrels were also included). Digital scans of the drawings were imported into a computer and area measures (mm^2^) acquired with NIH ImageJ software .

### Statistics

Analysis was done in the Stata/SE 8.2 statistics, graphics, and data management software package , and consisted of graphic plots and descriptive statistics followed by inferential statistics. We used analysis of variance (ANOVA) and analysis of covariance (ANCOVA) to test differences in means between strain, and logistic regression models to predict strain identity. Diagnostic plots and statistics were investigated to validate model assumptions in each case. Alpha was set to 0.05 for statistical significance. Asking if there is a difference in cortical field size between strains when the field correlates with total neocortex size, it is reasonable to consider forming a relative index of size, such as a proportion or percent (ratio) measure. Use of a ratio measure may fail to control brain size [27]. An alternative to forming a ratio is to use ANCOVA, which adds a covariate to an ANOVA model to statistically control that covariate. For strain prediction, we chose logistic regression rather than discrminant analysis. Both methods produce the same prediction table with our data, while logistic regression carries fewer assumptions and is likely more familiar to the reader. The projection of a rotating plot for neocortex, V1, and PMBSF, was produced in the Data Desk 6.2 statistics, data mining, and visualization software package .

## Authors' contributions

DCA and CEC conceived and designed the study. DCA, CEC, AIR, KME, and FW collected data. DCA analyzed the data and prepared the manuscript.

## Figures and Tables

**Table 1:** Prediction table for strain identity. This table shows actual and predicted classification of strain from a logistic regression model predicting C57BL/6J and DBA/2J from neocortex, visual cortex, and barrel cortex area.

Logistic regression confusion matrix			
	Predicted		

Actual	DBA/2J	C57BL/6J	Total
DBA/2J	15	1	16
C57BL/6J	2	12	14
Total	17	13	30
